# Sphingolipid-Induced Bone Regulation and Its Emerging Role in Dysfunction Due to Disease and Infection

**DOI:** 10.3390/ijms25053024

**Published:** 2024-03-05

**Authors:** Anouska Seal, Megan Hughes, Fei Wei, Abinaya S. Pugazhendhi, Christopher Ngo, Jonathan Ruiz, Jonathan D. Schwartzman, Melanie J. Coathup

**Affiliations:** 1 Biionix Cluster, University of Central Florida, Orlando, FL 32827, USA; lilyseal205@gmail.com (A.S.); fei.wei@ucf.edu (F.W.); abinayasindu.pugazhendhi@ucf.edu (A.S.P.); christopher.ngo@ucf.edu (C.N.); 2 School of Biosciences, Cardiff University, Cardiff CF10 3AT, UK; meganjh5@aol.com; 3 College of Medicine, University of Central Florida, Orlando, FL 32827, USAjo535595@ucf.edu (J.D.S.)

**Keywords:** sphingolipids, bone, infection, disease, osteoporosis

## Abstract

The human skeleton is a metabolically active system that is constantly regenerating via the tightly regulated and highly coordinated processes of bone resorption and formation. Emerging evidence reveals fascinating new insights into the role of sphingolipids, including sphingomyelin, sphingosine, ceramide, and sphingosine-1-phosphate, in bone homeostasis. Sphingolipids are a major class of highly bioactive lipids able to activate distinct protein targets including, lipases, phosphatases, and kinases, thereby conferring distinct cellular functions beyond energy metabolism. Lipids are known to contribute to the progression of chronic inflammation, and notably, an increase in bone marrow adiposity parallel to elevated bone loss is observed in most pathological bone conditions, including aging, rheumatoid arthritis, osteoarthritis, and osteomyelitis. Of the numerous classes of lipids that form, sphingolipids are considered among the most deleterious. This review highlights the important primary role of sphingolipids in bone homeostasis and how dysregulation of these bioactive metabolites appears central to many chronic bone-related diseases. Further, their contribution to the invasion, virulence, and colonization of both viral and bacterial host cell infections is also discussed. Many unmet clinical needs remain, and data to date suggest the future use of sphingolipid-targeted therapy to regulate bone dysfunction due to a variety of diseases or infection are highly promising. However, deciphering the biochemical and molecular mechanisms of this diverse and extremely complex sphingolipidome, both in terms of bone health and disease, is considered the next frontier in the field.

## 1. Introduction

Lipids are fundamental building blocks ubiquitous in all cells and are increasingly becoming recognized as critical components of major signaling and regulatory pathways in human biology, physiology, and pathophysiology. Remarkably, the eukaryotic lipidome is comprised of an extremely heterogeneous body of molecules, and emerging analyses reveal unprecedented and unanticipated complexity, whereby many stimuli and species affect one or more enzymes [1]. Due to the large number of varying biochemical transformations that occur during their biosynthesis, lipid structures are generally much more complex than linear protein combinations [2]. As such, lipid diversity is considered of a magnitude that contends with the proteome, where together, cells express tens of thousands and hundreds of lipids and proteins, respectively, together tightly regulating metabolism, energy storage, and transport [2,3]. Using the LIPIDs MAPS classification system, lipids have been divided into eight categories: fatty acyls, glycerolipids, glycerophospholipids, sphingolipids, sterol lipids, prenol lipids, saccharolipids, and polyketides, with each containing distinct classes and subclasses of biomolecules, thereby delivering differing effects [2,4,5].

The concept of bioactive lipids and their role in human physiology and pathophysiology has increasingly evolved over recent decades and continues to gain considerable traction [6]. Bioactive lipids are functionally defined as lipid species able to react and respond following the delivery of specific stimuli, thereby in turn mediating definitive downstream effectors and targets via their contribution to various signaling pathways [7,8]. As such, bioactive lipids are distinguishable from other lipids with known structural and/or energetic functions. Recent research has shown that sphingolipids, a major class of eukaryotic lipids containing 4000 distinct chemical entities [9], are highly bioactive and activate distinct protein targets, including lipases, phosphatases, kinases, and other membrane receptors and enzymes, thereby confirming distinct cellular functions beyond energy metabolism [1,10]. In this respect, sphingolipids have been shown to be critical in mediating cellular processes, including regulating the actin cytoskeleton, endocytosis, cell cycle, apoptosis [11], and cell stress responses, including programmed cell death [12], senescence [13], cell survival, migration, adhesion, inflammation, vesicular trafficking, and phagocytosis [1,14,15,16]. Although sphingolipids represent a small proportion of the total cellular lipid pool, their aberrant ectopic accumulation within tissues not equipped for fat storage is reported to drive cellular dysfunction and damage [17,18]. To this end, sphingolipids have been theorized to contribute essential roles in cancer [19], obesity [20], type 2 diabetes, cardiovascular and immune dysfunction, Gaucher’s disease, and neurodegeneration [1,18,21,22]. However, and despite being a rapidly expanding area of high significance, their potential role as biomarkers of disease, diagnostic markers differentiating between bacterial and viral infections, or use towards the development of novel therapeutic approaches remains elusive. To this end, a major unresolved challenge is the necessity to identify and decode their structures and biochemical and molecular mechanisms of action.

### 1.1. A Summary of Eukaryotic Sphingolipid Metabolism

Sphingolipids are ubiquitous and involved in a diverse number of cell functions, making them essential components within all eukaryotic membranes [1]. The synthesis and metabolism of sphingolipids are not presented here but have been previously and comprehensively reviewed [1,20,21,23,24,25,26]. In brief, sphingolipids are characterized by the presence of a long-chained sphingoid base together with a 2-amino group amide linked to a fatty acid (FA) and a polar head group. This combination forms ceramide, the core unit. As such, ceramides constitute a family of closely related molecules and form the precursor and metabolic hub of all complex sphingolipids. In brief, ceramide occupies a central position within a highly coordinated system that interconnects several pathways in both sphingolipid biosynthesis and catabolism. Fundamentally, the family of sphingolipids is defined by the type of FA, carbon length, polar head group, degree of unsaturation, and hydroxylation, among other characterizations [10].

**Figure 1:**
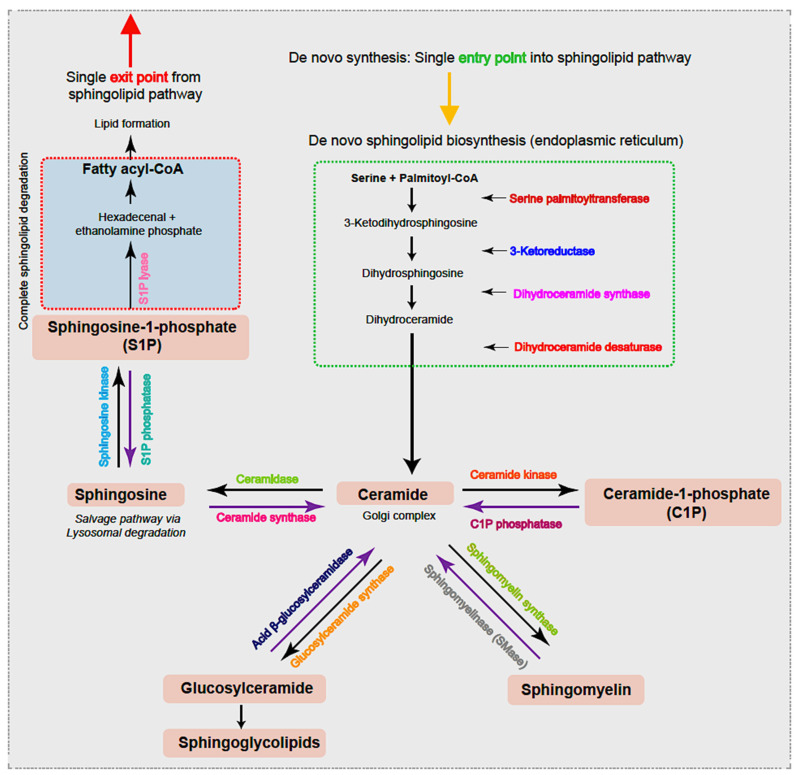
Ceramide, the hub of the sphingolipid pathway, can be generated via different pathways. De novo synthesis, i.e., the single entry point into the cycle, begins in the ER with condensation of typically serine and palmitoyl-CoA to form 3-ketosphingosine. The 3-ketodihydrosphingosine is subsequently and rapidly converted to dihydroxyceramide prior to acylation to dihydroceramides by the action of dihydroceramide synthase. Dihydroceramide is then dehydrated by dihydrodesaturase (DES) to form ceramide, which is translocated to the Golgi complex. Ceramides can also be generated via the sphingomyelinase (SMase) pathway that degrades sphingomyelin or via the catabolic pathway that generates ceramides from sphingosine by ceramide synthase from glucosylceramides by acid β-glucosylceramidase or by C1P via the action of C1P phosphatase. Similarly, and in parallel, the reactions are reversible, and ceramides generate sphingomyelin through the activity of sphingomyelin synthase, sphingosine through the enzyme ceramidase within the lysosome, and C1P via ceramide kinase activity. Further, sphingosine can instead be converted to S1P via sphingosine kinase or instead irreversibly cleaved by ER-localized S1P lyase, leading to complete sphingolipid degradation, the single exit route within the sphingolipid pathway. The various enzymes involved within the pathway are highlighted in color.

The first step in the de novo sphingolipid biosynthetic pathway begins in the endoplasmic reticulum (ER) with condensation of an amino acid, most frequently serine, typically together with the 16-carbon saturated FA palmitate, which is activated following a reaction with fatty acyl-CoA [20] (Figure 1). This reaction is catalyzed by the enzyme serine palmitoyltransferase (SPT), a membrane-bound component of the ER, to form transient 3-ketodihydrosphingosine. The 3-ketodihydrosphingosine is then rapidly reduced to dihydrosphingosine followed by *N*-acylation by one of six ceramide synthases to form dihydroceramides [27,28]. Dihydroceramide desaturase (DES1 or DES2) subsequently yields ceramide, as shown in Figure 1 [29,30]. Notably, as the enzyme SPT is the only entrance point into the sphingolipid network, it uniquely serves as a critical node for regulating the steady state and rate-limiting fluctuations in sphingolipid metabolism [31]. To this end, the abundance of the sphingolipid pool is primarily dictated via feedback from a family of three highly homologous ER membrane-bound ORMDL proteins. These homeostatic regulators of SPT are able to sense ceramide levels, and when beyond physiologic thresholds, the ORMDL proteins subsequently inhibit SPT activity [20,31,32]. Notably, another protein has also been found essential in regulating SPT activity. A more recent investigation highlighted the important contribution of Nogo-B, a membrane protein of the ER, which also contributes direct effects to SPT activity [33]. However, its interactions with both SPT and ORMDL proteins remain poorly understood.

Following both vesicular- and protein-mediated transportation from the ER and predominantly within the Golgi complex, ceramides serve as a substrate for sphingolipid metabolism. Through modifications located at the 1-hydroxyl position, ceramides are incorporated into various complex sphingolipids that fall within four primary categories. First, ceramide phosphorylation occurs via the enzyme ceramide kinase and generates the sphingolipid ceramide-1-phosphate (C1P) [34]. In the second category, the addition of a sugar molecule leads to glycosylation by glucosyl or galactosyl ceramide synthases to form glycoceramides and, subsequently, glycosphingolipids [35]. Third, and via sphingomyelin synthases, ceramides can receive a phosphocholine head group from phosphatidylcholine to form sphingomyelin (SM), the most abundant mammalian sphingolipid [1,36]. The fourth category is formed within the lysosome where the acyl chain may be removed from the ceramide substrate to produce the lyso-sphingolipid sphingosine and via action of the enzyme ceramidase [37]. Notably, sphingosine can be salvaged and recycled to ceramide for reutilization by the action of ceramide synthase or, alternatively, phosphorylated by sphingosine kinases (SPHK1 and SPHK2) to form sphingosine-1-phosphate (S1P). S1P can then be irreversibly cleaved by ER-localized S1P lyase, forming the single exit route, a pathway that results in complete sphingolipid degradation. Alternatively, S1P can be dephosphorylated by S1P phosphatase and returned to form sphingosine followed by ceramide. Notably, ceramide (proapoptotic) and S1P (antiapoptotic) display opposing effects, and it has been suggested that the ratio of these two molecules is crucial to understanding their pathological roles [20]. Further, SM, C1P, and glucosylceramides can also be salvaged and recycled to ceramide for reutilization via the action of sphingomyelinase (SMase), C1P phosphatase, and acid β-glucosylceramidase activity, respectively.

Together, these reactions regulate extra- and intra-cellular sphingolipid concentrations prior to their being shipped to other membranes, where complex cellular trafficking subsequently directs their distribution to initiate a myriad of signaling pathways [18,38]. Thus, the sphingolipidome is highly complex, where each can interconnect and interconvert one bioactive lipid into others. Thus, functionally distinct pools of chemically equivalent sphingolipids may be generated by either de novo synthesis or by the recycling and reutilization of existing complex sphingolipids. It is highly conceivable that even minor alterations in the species or enzyme produced can result in a ripple effect, thereby dictating subsequent metabolite levels, enzyme activities, and specific cellular end-functions [39].

### 1.2. The Basics of Healthy Bone Homeostasis

**Figure 2:**
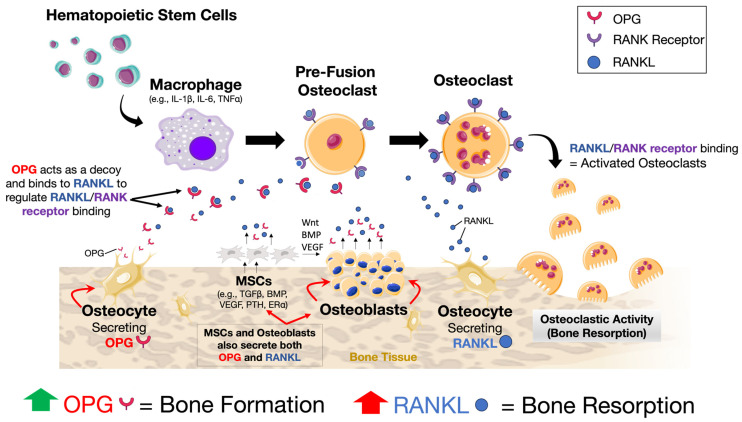
A schematic showing the relationship between RANK, RANKL, and OPG in activating or inhibiting osteoclastic activity and subsequent bone resorption. The binding of RANKL to the membrane-bound receptor RANK results in the activation of osteoclastic activity. However, OPG acts as a decoy and, if present, will bind to RANKL, thereby preventing RANK–RANKL interaction and osteoclastic activation. Thus, bone formation versus resorption is fundamentally dependent on the RANKL:OPG ratio, where increased RANKL expression results in bone resorption and increased OPG in bone formation. Many cells secrete either or both OPG or RANKL or both (e.g., osteocytes, osteoblasts, BMSCs, B-lymphocytes, and megakaryocytes). Regulation of the cellular response occurs via mechanical, hormonal, and growth factor-induced signaling, among others, making the overall governance of this system highly complex.

Bone is a highly dynamic and metabolically active tissue. Throughout life, it undergoes synchronous events that together orchestrate and tightly regulate cycles of bone resorption and formation. When in balance, this essential sequence of events enables the repair, restoration, and maintenance of a healthy bone tissue microenvironment, as well as the integrity of a mechanically functional structure. Traditionally, the critical cells involved include bone-forming osteoblasts and their precursors (i.e., bone marrow-derived mesenchymal stem cells (BMSCs)), bone-resorbing osteoclasts and their precursor cells, and the overarching regulatory contribution of osteocytes. In brief, bone formation or resorption occurs through complex interactions between (i) the transmembrane receptor activator of nuclear factor-κB (NF-κB (also known as RANK)), (ii) the receptor activator of nuclear factor κB ligand (RANKL), and (iii) the decoy protein osteoprotegerin (OPG), which together upregulate or downregulate osteoclast activity (Figure 2) [40,41,42,43]. Fundamentally, the up- or downregulation of bone formation or resorption is maintained via fluctuations in the RANKL:OPG ratio, where increased RANKL predisposes to increased osteoclastic activity, while a higher abundance of OPG is associated with increased osteoblastic activity and bone formation. Furthermore, emerging studies highlight the important osteoimmunological contribution. For example, macrophages directly regulate bone turnover through release of either pro-inflammatory cytokines (e.g., interleukin 1β (IL-1β) and IL-6), resulting in bone loss, or anti-inflammatory cytokines (e.g., IL-10 and IL-13) that promote bone formation and repair [44,45,46]. Further, mature B-lymphocytes produce >50% of total bone marrow-derived OPG, and mice lacking both T- and B-lymphocytes develop osteoporosis, although the critical role of T-cells in bone homeostasis is less clear [47,48]. Further, megakaryocytes derived from hematopoietic cells express both RANKL and OPG, and secrete anti-osteoclastic and bone anabolic factors [49,50]. Mechanical signals (e.g., compression, tensile strain, and shear stress), as well as hormonal cues (e.g., calcitonin, parathyroid hormone, 1,25(OH)_2_ vitamin D_3_, and estrogen) and growth factors (e.g., insulin-like growth factor (IGF), transforming growth factor beta (TGFβ), fibroblast growth factors (FGFs), epidermal growth factor (EGF), Wingless-related integration sites (Wnts), and bone morphogenetic proteins (BMPs)) are critical activators of bone formation or loss [51,52,53]. Also among the essential mechanisms for healthy bone homeostasis is the coordinated migration and trafficking of cells between the bone marrow and blood, as well as the regulated migration of precursor and mature cells to distinct regions on the bone surface for remodeling or repair. However, the intrinsic spatial, biomolecular, and mechanotransduction complexities are nevertheless still debated [52].

### 1.3. Role of Lipids in Bone Turnover

Lipids are an important nutrient and contribute a critical component to bone homeostasis, regeneration, and bone disease. In terms of homeostasis, adipocytes regulate bone formation via the inhibition or promotion of osteoblast and osteoclast differentiation [54]. This is achieved in part through the expression and secretion of peptides derived from white adipose tissue, including leptin, adiponectin, omentin-1, vesfatin, and resistin, as well as via adipocytokines such as tumor necrosis factor alpha (TNFα), IL-6, and IL-1β [55,56,57,58]. Fatty acids [59,60], cholesterol [61,62], phospholipids [63], and several endogenous lipid metabolites, including prostaglandins [64] and oxysterols [65], are also reported to stimulate bone cell function and activity. The lineage commitment of BMSCs towards either an osteoblastic or adipogenic fate are closely related, and preferential engagement along one axis over the other contributes an essential role in regulating bone mass [54]. This is likely due to several extracellular signaling proteins possessing overlapping functions, thereby contributing a key role in determining adipogenic versus osteogenic fate. These include the master adipogenic transcription factor peroxisome proliferator activated receptor gamma (PPARγ), osteoblast-specific transcription factors Runx2 and Osterix, as well as other proteins, including BMP-2, BMP-4, BMP-6, BMP-7, BMP-9, IGF, and FGFs [54,66]. It has been generally assumed that lipids are present within the bone marrow only and are largely absent from the mineralized bone structure itself. However, evidence suggests the mineral component contains small amounts of lipids that may play a pivotal role in bone physiology [67]. In this respect, lipids are not only located within bone cells as loosely bound and easily utilized biomolecules but also form tightly associated complexes with proteins and minerals within the mineralized tissue matrix. The presence of lipid within the porous compartments of trabecular bone is reported to restrict its hydraulic permeability, thereby influencing the transport of nutrients and waste products and thus the metabolic function of the cells located within [68]. Further, van Gastel et al. [69] showed that the decreased availability of extracellular lipids resulted in chondrogenic over osteogenic differentiation of skeletal progenitor cells.

Our interest in the role of lipids in bone turnover is increasingly expanding due to recent evidence that indicates a reciprocal relationship between bone mass and bone marrow adiposity. To this end, an increase in bone marrow adiposity has been observed in most bone loss conditions, including aging [55,70], osteoarthritis [71,72], obesity [73], osteomyelitis [74], and various other pathological conditions [55,75,76,77]. Further, adipokines contribute to the progression of chronic inflammation [78], which is also a key cause of multiple pathological bone loss conditions [79,80,81,82,83]. Together, this is of high significance, as our aging population and obesity continue to rise globally. As such, the treatment and care of conditions, including osteoporosis, osteoarthritis, and other chronic inflammatory-associated health conditions, will undoubtedly bear a huge health–economic burden in all regions of the world [84,85,86]. Obesity plays an important role in immunity and is considered a state of prolonged inflammation. Recent clinical and experimental studies have determined a correlation between the progress of obesity and some infections, prompting emergence of the “infectobesity theory” [87]. As such, bacterial-targeted adipose dysfunction and use as a reservoir for pathogens could promote virulence, exacerbate infection, and ultimately result in bone loss. To this end, a switch in lineage commitment toward adipogenic differentiation at the expense of osteoblastogenesis and increased bone marrow adiposity has also been identified during bacterial infection [74,88]. Notably, bacteria including *Staphylococcus aureus* and *Escherichia coli* are able to adhere and internalize within adipose-derived MSCs, and the subsequent up- or downregulation of osteogenic versus adipogenic differentiation was observed to be dependent on the type of bacterial strain [89]. Finally, exposure to ionizing radiation (IR) is critical during radiotherapy but does cause osteoradionecrosis, osteoporosis, pathologic insufficiency fractures, and nonunions, causing significant pain and morbidity in cancer survivors [90,91,92,93]. A switch in lineage commitment toward adipogenic differentiation at the expense of osteogenic differentiation, thereby leading to increased bone marrow adiposity, is reported following IR-induced bone injury [65]. Additionally, the transdifferentiation of osteoblasts into adipocytes [66] has also been identified. Together, these studies highlight the important role of the osteo-adipogenic axis in bone disease and infection, conditions that currently affect a substantial number of individuals and for which effective treatment strategies remain to be discovered.

To date, it remains unclear whether the increase in marrow adiposity is a result of or the cause of bone loss [54]. Notably, osteoblasts produce and secrete various forms of lipids (e.g., triglycerides, cholesterol, and phospholipids [94,95]), as well as initiate the nonenzymatic oxidation of lipoproteins, which results in a reactive oxygen species-induced inflammatory microenvironment and osteopathogenesis [96,97,98]. Of the numerous classes of lipids that accrue, sphingolipids are considered among the most deleterious. Here, we review and summarize evidence that dysregulation of sphingolipid metabolism, in particular, ceramide, C1P, S1P, acid sphingomyelinase, and SM, correlates with the pathogenesis of bone disease, osteoporosis, and dysfunction due to bacterial infection.

## 2. Sphingolipids in Bone Homeostasis and Disease

### 2.1. Ceramides

**Figure 3:**
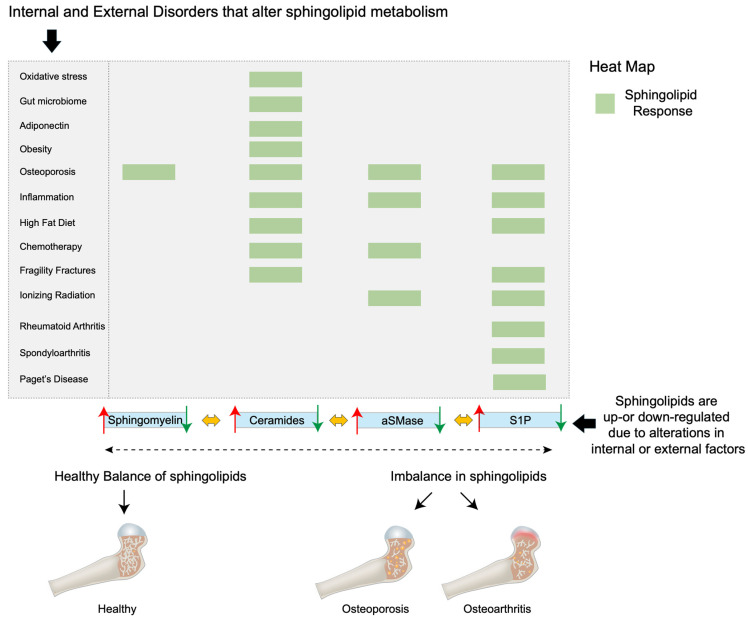
A heat map showing how biomolecules, the gut microbiome, diet, disease, and external factors (e.g., chemotherapy and ionizing radiation) can alter sphingolipid metabolism. When in balance, bone tissue function and structure are maintained. However, when sphingolipid levels, including sphingomyelin, ceramide, aSMase, and S1P, are either up- or downregulated, this can result in, for example, osteoporosis, fragility fractures, inflammation, Paget’s disease, osteoarthritis, rheumatoid arthritis, and spondyloarthritis.

Ceramides are signals of lipid excess and have been reported to confer many pathological effects. For example, ceramides promote the expression of genes that facilitate the incorporation of free FAs into harmful triglycerides and expedite their storage in lipid droplets [29,99]. Additionally, ceramides inhibit the uptake of both glucose [100,101] and amino acids [102,103], leading to the preferential utilization of FAs for energy. Further, ceramides decrease mitochondrial efficiency, inhibiting oxygen consumption via the electron-transport chain, thereby leading to decreased adenosine triphosphate (ATP) produced per FA molecule [104,105]. Finally, ceramides slow lipolysis by blocking activation of hormone-sensitive lipase [29]. Together, these ceramide actions promote the use and storage of FAs. Remarkably, preclinical rodent studies have shown that the inhibition of ceramide biosynthesis resulted in the amelioration of hypertriglyceridemia [29,106,107], type 2 diabetes [106], insulin resistance [29,106], hepatic steatosis [29,106,107], atherosclerosis [108,109], and heart failure [110,111]. It is now apparent that several metabolic disorders, including inflammation, oxidative stress, hormonal triggers, and the gut microbiome, influence ceramide synthesis and degradation [20]. To this end, ceramide production is elevated on exposure to excessive ROS [112], pro-inflammatory cytokine release [113,114], and alterations in gut bacteria of the phylum Bacteroidetes [115] (Figure 3). Further, elevated ceramide levels induce BMSC, fibroblast senescence, and death [13,116]. In the context of bone tissue, together, oxidative stress, pro-inflammatory cytokine release, and an altered gut microbiome are primary drivers associated with promoting bone resorption while impairing bone formation, thereby progressing the pathogenesis of bone disease and osteoporosis [117,118,119,120,121]. However, the direct effect of ceramide on bone remains largely unexplored.

At the cellular level, lower ceramide concentrations (≤10^−7^ M) are reported to promote osteoblast viability; however, levels ≥ 2 × 10^−6^ M significantly reduced viability and increased apoptosis in a dose- and time-dependent manner [122]. Alsahli et al. [123] investigated the osteoblast response to increased concentrations of palmitic acid, and similarly reported that ceramide accumulation inhibited osteoblast function in vitro and bone formation markers in a high-fat diet murine model in vivo. Further, endogenous cellular ceramide concentrations have been demonstrated to increase osteoblastic apoptosis following TNFα treatment and via the NF-κβ pathway [124]. Nevertheless, physiological concentrations of ceramide appear to be essential in bone development. Cilia, the mechanosensory antenna on many cell types, are present on osteoblasts and their precursors are dependent on the sphingolipid ceramide for their genesis [125]. Primary cilia are required for osteoblast and osteocyte polarity and alignment during bone development [126,127], and ciliopathies result in various skeletal abnormalities, including dysplasias [128,129]. Notably, ceramide depletion via neutral sphingomyelinase 2 (nSMase2), abolished primary cilia distribution and differentiation [130], and ceramide located at the base of cilia were shown to be critical in maintaining bone mass via β-catenin signaling in osteoblasts [127].

When stimulated, BMSCs mobilize from the bone marrow into the peripheral bloodstream and towards the site of bone injury, where they assist in regeneration and repair. The migratory “homing” of BMSCs is facilitated by factors including C1P [131,132], stromal cell-derived factor-1 (SDF-1) [133,134], substance P (SP) [135,136], and TGFβ [137]. A recent study by Yu et al. [138] demonstrated that intracellular ceramide kinase is also essential for BMSC migration for this purpose. Similarly, ceramides contribute to bone resorption, and the influence of ceramide on osteoclast apoptosis was also demonstrated to occur in a dose- and time-dependent manner [122]. To this end, ceramides have been demonstrated to mediate pro-apoptotic pathways and an anti-inflammatory response [6,139] while enhancing osteoclast survival and reducing osteoclastic activity via inhibition of F-actin ring formation [140]. Contrarily, increased levels of lactosylceramide, a glycosphingolipid, increased RANKL expression in osteoclasts, and data suggested that lactosylceramide is necessary for the initiation step of RANKL-induced osteoclastogenesis [141]. Several pro-inflammatory cytokines, including IL-1, increase cellular ceramide levels [6], and TNFα has also been shown to activate sphingomyelinases, thereby acutely liberating ceramides [12,142,143]. In terms of IL-1 activation, studies on hepatocytes have suggested that the ceramide formed is generated following the degradation of SM via the activation of neutral or acidic SMases to IL-1 [144,145]. Further, emerging evidence indicates that alterations in ORMDL protein levels confer a regulatory mechanism for the formation of sphingolipids. As described above, it has been widely considered that ORMDL proteins are negative regulators of SPT, where increased expression of ORMDL protein would further inhibit de novo sphingolipid synthesis [31]. However, and notably, overexpression of ORMDL3 increased SPT activity and ceramide levels in RAW264.7 macrophages, which promoted chronic inflammation in vitro and within a murine model in vivo [146]. Further, sphingolipid signaling during an IL-1-mediated inflammatory response has also been shown to increase ceramide levels via ORMDL protein expression during the de novo sphingolipid biosynthesis pathway [147]. Together, these data suggest a key concentration-dependent role for ceramides in driving both bone formation and bone resorption in bone metabolism.

Furthermore, human plasma levels of ceramide have been shown to increase with age in women, and are negatively correlated with serum estradiol concentrations [148]. To this end, Kim et al. [149] reported that ceramides directly increased osteoclastogenesis, the expression of osteoclast differentiation markers, and bone resorption in vitro. Further, the study also demonstrated increased levels of ceramide within the peripheral bloodstream of patients of increasing age. Notably, patients who presented with higher levels of ceramide were predisposed to increased fragility fractures of the hip and presented with increased bone resorption markers within blood and bone marrow aspirate biopsies. Notably, the de novo synthesis of ceramide and subsequent cellular apoptosis is enhanced in response to some chemotherapeutic agents, including etoposide and daunorubicin [150,151]. Finally, C1P, a product of ceramide phosphorylation, is widely reported to be proinflammatory via the NF-κβ pathway [152]. More recently, C1P was also found to potently activate cell growth and survival and regulate cell migration, as well as deliver anti-inflammatory properties to some cell types and tissues [153,154,155]. Additionally, C1P plays an important role in phagocytosis and macrophage chemotaxis [15,156].

### 2.2. Sphingosine-1-Phosphate

**Figure 4:**
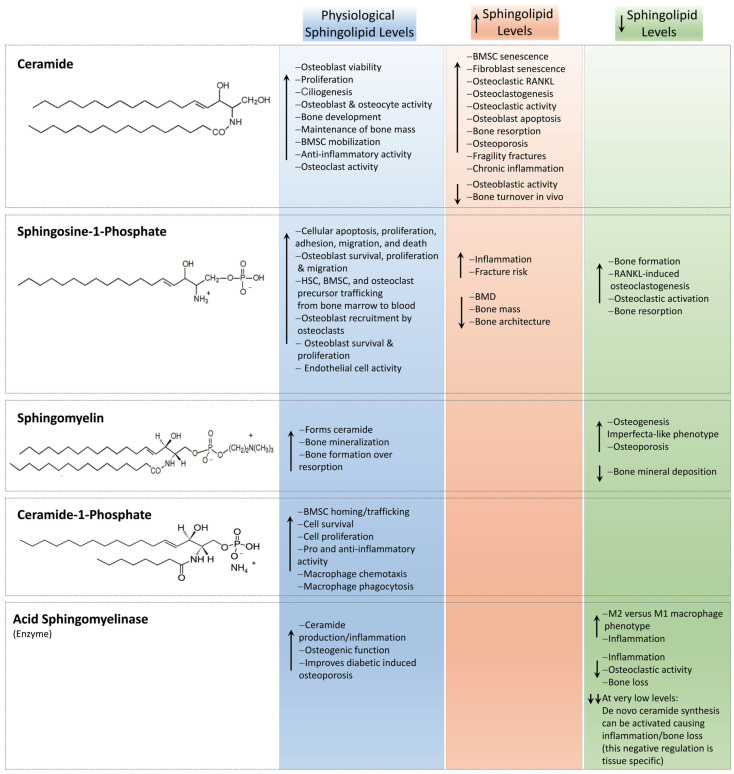
A schematic demonstrating the known effects of ceramides, C1P, sphingomyelin, aSMase, and S1P to the key cells within bone. The effect of physiological, increased, and decreased levels of each sphingolipid are described. Representative chemical structures are presented. The ↑ arrow indicates sphingolipid, biomolecule, cellular, or tissue upregulation, while the ↓ arrow highlights their downregulation.

The lipid mediator S1P is the most studied and can act directly on intracellular targets or alternatively via an intracellular second messenger or extracellular signal molecule by binding membrane receptors called G protein-coupled receptors, namely, S1PR_1_ to S1PR_5_ [67]. As such, S1P has both intracellular, S1PR-independent actions (e.g., calcium release [155], TNFα signaling [157], or PPARγ [158]) and S1PR-dependent extracellular functions in a variety of tissues. S1P has been shown to serve as a key mediator in the regulation of cell apoptosis [159], proliferation [160], migration, and death [161], as well as cell adhesion, motility, and platelet aggregation [162] in a variety of cells. In terms of bone tissue, S1PR_1_, S1PR_2_, and SIPR_3_ are predominantly expressed by mature osteoblasts [163], osteoclast precursors [164], and MSCs [165]. Sphingosine-1-phosphate is enriched within circulating blood compared with bone marrow and is reported to regulate hematopoietic stem cell (HSC) and osteoclast precursor migration between the bone marrow and peripheral blood stream [166]. The inhibition of S1P degradation or downregulation of S1PR_1_ by S1P lyase has been shown to dissipate the S1P gradient between blood and bone marrow and subsequently reduces the number of circulating HSCs [167]. Here, and at low S1P concentrations (<10^−7^ M), osteoclast precursors, as modeled using RAW 264.7 macrophages, expeditiously migrated towards the S1P chemoattractant in vitro, while at higher concentrations (10^−6^ M), S1P led to the chemorepulsion of macrophage migration, resulting in stationary cells [168]. This suggests the S1P gradient is important in osteoclast precursor trafficking to the bone surface, where they have been shown to undergo cell fusion to form terminal differentiated osteoclasts [164]. Similarly, BMSCs also display a dose-dependent migratory response to S1P. Using a Transwell migration assay, Kong et al. [169] showed that human-derived BMSCs migrated towards low concentrations (1–10 nM) of S1P, whereas no migration was measured when investigated at higher concentrations (50–1000 nM). Notably, Golan et al. [170] demonstrated that S1P induced SDF-1 secretion from BMSCs, which occurred via ROS signaling. Further, S1P has been shown to stimulate BMSC cell chemotaxis via Janus kinase (JAK)/signal transducers and activators of transcription (STAT) and focal adhesion kinase (FAK)/phosphatidylinositol 3-kinase (P13K)/protein kinase B (AKT) signaling and through both S1P_1_ and S1P_2_ [165]. However, the migratory BMSC response likely differs depending on whether downstream signaling is supported by S1PR_1_, S1PR_2_, or S1PR_3_ [165,169]. S1P has also been reported to be secreted by osteoclasts, where it promotes survival [171], and osteoblasts, predominantly through ATP-independent spinster 2 transporter activity [172,173]. Further, it has been reported that through S1P secretion, osteoclasts recruit osteoblast precursor cells and promote osteoblast survival, proliferation [163,174,175], and migration [176] via Wnt/BMP [177] and mitogen-activated protein kinase (MAPK) signaling [178]. Further, the generation of S1P is associated with the activity of 17β-estradiol, and both S1P and estrogen are reported to augment osteoblast proliferation [174]. In the study, Tantikanlayaporn et al. reported that estrogen increased SPHK1 protein expression in human osteoblasts, and notably, S1P upregulated estrogen receptor (ER)-β mRNA expression, but not ERα, as well as increased levels of SPHK1 and S1PR_1_. Keller et al. [172] investigated S1P via the bone resorption inhibitor calcitonin and revealed S1P as an osteoanabolic molecule. In support of this, Weske et al. [179] increased S1P levels through the inhibition of S1P lyase in a murine model. Notably, data showed decreased levels of white adipose tissue formation alongside increased bone formation, mass, and strength. Further, Ishii et al. [164] showed that in a murine model, daily injection of the nonselective S1P receptor agonist FTY720 (fingolimod; 2-amino-2-[2-(4-octylphenyl)ethyl]propane-1,3-diol hydrochloride), a novel immunosuppressive drug, reduced ovariectomy-induced osteoporosis. The study confirmed a decrease in the number of mature osteoclasts in contact with the bone surface, suggesting the potential of S1P as a therapeutic agent for osteoporosis. Conversely, S1P has also been shown to augment osteoclastogenesis. All S1P receptors with the exception of S1PR_5_ have been detected in bone marrow-derived macrophages and differentiating osteoclasts [171,172]. Ryu et al. [171] investigated the effect of S1P on the osteoclastogenesis of bone marrow-derived macrophages. The study showed that RANKL upregulated sphingosine kinase activity, thereby increasing intracellular S1P production and secretion, with no osteoclastogeneic differentiation measured. In contrast, the addition of S1P to a co-culture of osteoblasts and bone marrow-derived macrophages significantly increased osteoclastogenesis via increased COX-2 and PGE_2_ and, subsequently, RANKL production in osteoblasts and T cells. Further, S1P was also demonstrated to activate osteoblast migration and survival, together suggesting that secreted S1P attracts and activates both osteoblasts and T cells to augment osteoclastogenesis, but appeared to have no direct effect on bone marrow-derived macrophage osteoclastogenesis itself. To this end, Xiao et al. [180] unveiled the regulatory role of the sphingosine kinase/S1PR_1_/RANKL axis in increasing inflammatory bone loss. The study showed that stimulated macrophages induced sphingosine kinase activity, which led to the activation of S1PR_1_ in BMSCs and the production of RANKL. Notably, S1PR_1_ blockage abolished this affect, and the authors proposed this cell-signaling pathway as a potential future therapeutic target. As such, S1P facilitates the proliferation, migration, and survival of osteoblasts, and couples osteoblast–osteoclast communication and induces RANKL-induced osteoclastogenesis, osteoclastic activation, and bone resorption (Figure 4) [171].

Notably, inflammation is reported to be associated with high levels of S1P in humans [181]. To this end, high plasma levels of S1P correlated with decreased bone mineral density (BMD) [181,182,183], bone mass and architecture [184], and parathyroid hormone levels [179,181], and a 9.33-fold [182] and 9.89-fold [183] increased risk of vertebral fracture in post-menopausal women. Further, incident fractures have also been reported to occur more frequently in post-menopausal women who presented with increased plasma S1P levels [185]. Importantly, Ardawi et al. [183] reported that plasma S1P levels and their association with fracture risk were independent of BMD and other established clinical risk factors. Further, Lee et al. [181] confirmed that high S1P plasma levels consistently identified as a significant risk factor in patients with osteopenia and that this was independent of the established fracture risk assessment tools developed by the World Health Organization. These data strongly support S1P as a new and independent biomarker for the risk of osteoporotic fracture [186], although the role of an S1P-tissue gradient (e.g., between bone marrow and plasma) also appears to be important when determining the fracture risk response [187,188]. It has been suggested that the major effect of S1P in regulating bone homeostasis involves the preferential recruitment of osteoclast precursors as opposed to the stimulation of an osteoanabolic response [181,184,185]. In this context, Grewe et al. [189] speculated that high S1P levels would result in the internalization of S1PR_1_, thereby resulting in the dominant expression of S1PR_2_ on the osteoclast precursor cell surface. This would subsequently drive the recruitment of osteoclast precursor cell migration into the bone marrow via S1PR_2_-induced chemorepulsion. However, a study by Heilamann et al. [190] found no improvement in fracture healing when FYT720 was administered daily for 10 and 21 days in a murine osteotomy model of bone fracture. Interestingly, Paget’s disease is characterized by an increased number of abnormal osteoclasts, which drive elevated levels of bone formation [191]. Notably, Nagata et al. [192] demonstrated significantly increased levels of SPHK1 secretion from osteoclasts isolated from a murine model of Paget’s disease as well as osteoclasts isolated from a human donor of the disease. The study identified a Paget’s disease-induced increase in IL-6 production by osteoclasts, subsequently increasing S1P secretion, which, together with IGF-1, promoted S1PR_3_ expression in osteoblasts to promote bone formation. The authors concluded that when combined, and via the upregulation of Ephrin (Eph) B2 and EphB4 in osteoclasts and osteoblasts, respectively, increased S1P via S1PR_3_ signaling resulted in the displayed increase in bone formation observed in Paget’s disease. The study concluded that S1P-based drugs may offer a promising anabolic treatment for bone loss.

The enhanced turnover of subchondral trabecular bone is a hallmark of rheumatoid arthritis (RA) [193]. To this end, and notably, significantly increased levels of S1P have been identified within the synovium of patients with RA (17.5 μM) compared to patients with osteoarthritis (3.5 μM) [194]. Notably, the inhibition of S1P has also been shown to alleviate osteoarthritis in a knockout Sphl1^LysMCre^ murine model [195]. As such, therapeutics that target S1P have been proposed for use in the elderly [196]. To this end, and in a murine model of collagen-induced RA, the pharmacological or siRNA knockdown of SPHK1 significantly reduced release of the inflammatory cytokines TNFα, IL-6, IL-1β, MCP1, and MMP9 in vitro [194]. Further, the study showed that joint erosion and serum levels of inflammation were also significantly reduced in vivo. In support of this, and in a rat collagen-induced RA model, administration of the S1PR agonist FTY720 inhibited the formation of synovitis and bone erosions more effectively than prednisone [197]. Further, and in both an adjuvant-induced and collagen-induced rat model of RA, FTY720 successfully inhibited joint inflammation to equal or higher efficacy compared to mizoribine and prednisone [198]. Hutami et al. [193], using a Fas-deficient MRL/lpr mouse model that spontaneously develops autoimmune arthritis and exhibits reduced bone mass, demonstrated significantly increased S1PR_1_ within the condylar cartilage of the temporomandibular joint. The study revealed that Fas/S1PR_1_ signaling via NF-κB was necessary for S1P-induced migration of osteoclast precursor cells and that inhibition of NF-κB resulted in the reduction of SPHK1/S1P_1_ signaling and subchondral bone loss. Finally, and as S1P is able to prevent IL-1β-induced cartilage degradation, Stradner et al. [199] demonstrated that the S1PR agonist FTY720 significantly reduced TNFα, IL-1β, and iNOS levels in chondrocytes in vitro. Together, these studies suggest that increased levels of S1P via SPHK1 contributes a primary role in the progression of inflammatory arthritis, and SPHK1 modulation may provide a novel approach to treating autoimmune conditions such as RA. Spondyloarthritis is a group of chronic rheumatic inflammatory diseases and the second most common type of inflammatory arthritis after RA [200,201]. Bougault et al. [202] recently reported that S1P serum levels were significantly elevated in spondyloarthritis patients (6.1 μM) compared to healthy individuals (1.6 μM). Interestingly, cyclic stretch has been shown to enhance *SPHK1* gene expression in cultured osteoblasts and chondrocytes, where supplementation with TNFα or IL-17 further increased stretch-induced *SPHK1* upregulation [203]. The authors speculated that S1P production by chondrocytes may therefore be stimulated by both inflammation and mechanical stress in spondyloarthritis.

Notably, S1P opposes the proapoptotic function of ceramide, and the ratio of S1P to ceramide has also been described as a sphingolipid rheostat involved in pathogenesis [204]. Notably, inducible overexpression of adiponectin receptors in adipocytes enhances ceramidase activity, thereby promoting ceramide to sphingosine [205]. Thus, targeting S1P and its receptors may represent a novel route to prevent or reduce osteoporosis. However, together, these studies indicate that S1P signaling is a highly dynamic interconnecting network that involves multiple players, and as such, is substantially complex. Therefore, further mechanistic clarity is first warranted. Nevertheless, sphingolipid-based therapies may hold significant promise in beneficially regulating diseases that cause disturbances where bone resorption exceeds bone formation.

### 2.3. Acid Sphingomyelinase

Mechanistically, sphingomyelinases function as hydrolases of phospho-diester bonds, where their peak activity is contingent on the local pH [206]. As such, sphingomyelinases are classified as acidic, neutral, or alkaline, and are located in separate cellular sub-locations, where the activity of their products mediates specific and targeted functions [207]. Acid sphingomyelinase (aSMase) is an enzyme that catalyzes the conversion of SM into ceramide, and its deficiency results in the lysosomal storage disease known as Niemann–Pick-type A and B [208]. aSMase activation is reported to increase ceramide production via the hydrolysis of SM, and this is associated with the amplification of inflammatory signaling in macrophages, as well as cellular stress signaling pathways [209,210]. In terms of bone tissue turnover, the inhibition of aSMase by imipramine ameliorated the synergy between metabolic syndrome and *Aggregatibacter actinomycetemcomitans*-induced periodontitis and alveolar bone loss in a high-fat diet-fed murine model [211]. Notably, the study showed that the inhibition of aSMase reduced ceramide production and subsequently lipopolysaccharide (LPS)-induced periodontitis by reducing pro-inflammatory and pro-osteoclastogenic gene expression in macrophages in vitro and metabolic syndrome-induced periodontitis in vivo. Based on these results, the authors suggested aSMase as a potential therapeutic target. Interestingly, a previous study by this group demonstrated that in a low-fat diet-fed murine model, aSMase-deficiency instead exacerbated LPS-induced periodontitis [212]. Remarkably, both the SM and ceramide content in mice increased with aSMase deficiency, and the unexpected increase in ceramide formation occurred via de novo synthesis, which was upregulated to compensate for the experimentally induced low levels of aSMase. In support of this, Deevsaka et al. [213] also reported increased ceramide de novo synthesis within the liver of aSMase-deficient mice. Notably, de novo ceramide synthesis was found to not occur in all tissues within aSMase-deficient mice, thereby suggesting that the negative regulation of ceramide de novo synthesis is tissue-specific [212]. Roux-Biejat et al. [214] reported that aSMase regulated the expression of macrophage M1 versus M2 phenotype and that the absence of aSMase reduced inflammation in a murine muscle model. Further, the long-term use of fluoxetine, a commonly prescribed antidepressant, is increasingly being associated with increased bone fragility. Interestingly, Zhang et al. [215] showed that through the inhibition of aSMase, fluoxetine induced the disruption of sphingolipid metabolism within bone marrow adipose tissue. In contrast, the study demonstrated that a significant reduction in bone volume was observed in aSMase knockout mice. The formation of ceramide via the de novo synthesis pathway was not investigated, and here, the authors speculated that the inhibition of aSMase reduced ceramide and S1P levels within bone marrow adipocytes, leading to RANKL secretion via cyclooxygenase-2 (COX-2) and its enzymatic product, prostaglandin E_2_ (PGE_2_), in a dose-dependent manner. The overproduction of PGE_2_ induced the secretion of RANKL, thereby promoting osteoclastogenesis. Remarkably, clinically administered oral supplementation of L-serine (250 mg/kg/d), a precursor of SPT, and thus sphingolipid de novo biosynthesis, prevented the fluoxetine-induced accelerated bone loss in postmenopausal women with major depressive disorder, thereby providing new insights and a potential future treatment strategy. Finally, ferroptosis, an iron-catalyzed form of regulated necrosis, has been implicated in the pathological process of type 2 diabetes-induced osteoporosis. Du et al. [216] showed that osteoblastic expression of aSMase and ceramide increased when in a high glucose environment. The study showed that an increase in aSMase levels improved osteogenic function by decreasing high glucose-induced autophagy, GPX4 degradation, and ferroptosis. The authors suggested that aSMase regulation may be a promising method for the treatment of diabetes-induced osteoporosis.

Further, multiple stress stimuli, including ionizing radiation and chemotherapeutic agents (e.g., platinum, paclitaxel, and histone deacetylase inhibitors), have been demonstrated to rapidly activate aSMase [6], potentially with the simultaneous generation of ceramide [217]. Notably, both chemotherapy (e.g., methotrexate, imatinib, and taxanes) and the exposure to IR can cause significant bone loss and fragility [218,219]. The mechanisms of how stress agents activate aSMase remain elusive. However, ROS has been shown to activate aSMase [220], and, for example, IR is known to significantly upregulate oxidative stress within bone tissue [79] as well as ceramide levels [221]. Endothelial bone cell crosstalk and vessel networks are critical in bone homeostasis and repair [222], and high-dose radiotherapy (>8–10 Gy) causes expeditious endothelial cell death via the hydrolyzation of SM to ceramide via aSMase [223,224]. In contrast, low doses (<6 Gy) are reported not to biologically activate ceramide production. Notably, the pre-treatment of endothelial cells with S1P has been shown to reduce ceramide-induced IR cell death, and aSMase knockout mice are more radioresistant to high-dose IR than healthy control animals [225,226]. At the cell membrane, ceramide molecules interact and form stable and tightly packed ceramide-enriched membrane domains. These domains can spontaneously associate to form large ceramide-enriched membrane macrodomains, also called lipid rafts, ~10–200 nm in size, increasing to >300 nm if they fuse to form microscopic domains via protein–protein and protein–lipid interactions [227,228,229]. Ladjohounlou et al. [229] recently demonstrated that ceramide-enriched platforms play a significant role in both the targeted and non-targeted “bystander” effects in head and neck squamous cell carcinoma cells following exposure to IR. The study reported that the preexistence of these domains led to cellular insensitivity to the non-targeted effects of IR and, depending on the genesis and/or intrinsic nature of the cells, ceramide-enriched domains may modulate both cell survival and/or death. Notably, S1P administration prior to IR exposure has been reported to prevent gastrointestinal syndrome by inhibiting endothelium collapse in mice following exposure to 10 Gy [230]. Further, Bonnaud et al. [231] demonstrated that S1P protected human microvascular endothelial cells from ceramide-induced apoptosis following 15 Gy of IR, but not from DNA damage-induced mitotic death. Most notably, opaganib, a first-in-class inhibitor of sphingolipid metabolism via SPHK2, has been shown to deliver broad anti-inflammatory (via the downregulation of NF-κB and TNFα) and anti-cancer activity [232]. As such, opaganib acts as a sphingosine mimetic molecule and also inhibits DES1, thereby increasing levels of dihydroceramides and promoting autophagy [233]. Opaganib is reported to elevate ceramide and reduce S1P in cells, thereby increasing the antitumor efficacy of IR while suppressing inflammation. To this end, mice exposed to high-dose IR showed a dose-dependent survival advantage following the oral administration of opaganib 4 h before or 24 h after radiation exposure. Remarkably, opaganib substantially protected normal host tissue from IR-induced damage.

Together, these studies highlight complexity in the function of aSMase and its influence on bone regulation, as well as the magnitude of aSMase inhibition and the important alterations that ensue in terms of the regulation and activation of de novo biosynthesis and subsequent opposing cellular and bone tissue responses. However, it remains a promising therapeutic approach to several conditions that negatively impact bone regeneration and repair.

### 2.4. Sphingomyelin

Sphingomyelin constitutes a major membrane component within, for example, lipid rafts, caveolae, and clathrin-coated pits, thereby contributing to the mediation of transmembrane signaling [234]. As such, SM has been indicated to play a key role in cell survival, proliferation, migration, and inflammation [36,234]. Nevertheless, its bioactivity is considered to mainly rely on its hydrolysis and the downstream formation of ceramide and S1P [10]. In situ SM hydrolysis within bone results in the formation of the bioactive lipid metabolites phosphocholine and ceramide, and both are critical for bone mineralization. In this respect, the mineralization of matrix vesicles, which bud from the plasma membrane of mineral-forming cells such as osteoblasts, chondrocytes, and odontoblasts [63], is associated with the rapid breakdown of SM, suggesting the involvement of a sphingomyelinase [235]. Notably, deletion of the gene *Smpd3*, which encodes nSMase2, a membrane-bound enzyme that hydrolyses SM to phosphocholine and ceramide, results in mice displaying a severe osteogenesis imperfecta phenotype [236,237]. Further, nSMase2 is reported to be highly expressed in mature osteoblasts, and cells deficient in this enzyme reveal an impairment in their ability to deposit minerals in vitro [238]. However, the mechanisms by which nSMase2 regulates bone mineralization remain to be determined. It is possible that via the generation of phosphocholine nSMase2 may participate in the local increase in the PO_4_^3−^ pool within bone, thus facilitating the initiation of mineralization [67]. Further, nSMase2 activity also generates ceramide, which can be further hydrolyzed to sphingosine, while both phosphocholine and ceramide can be further phosphorylated via kinases to C1P and S1P [239]. The SM-mediated mechanisms leading to osteoporosis are incompletely understood. To this end, a study by Pekkinen et al. [240] showed that six families with rare skeletal phenotypes were all identified with a heterozygous variant in the SM synthase 2 gene, suggesting a fundamental and critical role for SM metabolism in a spectrum of conditions ranging from osteoporosis to complex skeletal dysplasia. Further, SM synthase 2-knockout was demonstrated to suppress TRAP-positive osteoclast staining via RANKL in mice, suggesting the potential likelihood of a critical contribution towards bone formation rather than bone resorption [241]. Finally, and when loaded with cholesterol, SPT and SM levels were reported to increase in macrophages [242]. Notably, the study showed that ORMDL levels were reduced with cholesterol loading, thereby potentially reducing ORMDL-dependent SPT activity. However, the mechanistic role of cholesterol in sphingolipid metabolism remains to be elucidated.

## 3. Sphingolipids and Infection-Induced Bone Loss

Surgical site infections and periprosthetic joint infections (PJIs) associated with bone or bone marrow are extremely challenging complications that are coupled with high morbidity and the need for complex treatment strategies [243,244]. Biofilms, communities of surface-adherent microbes (e.g., bacteria) protected by a polymeric matrix, are notoriously difficult to treat. Their formation is associated with ~1.7 million hospital-acquired infections per year in the United States, incurring an annual economic burden of ~USD 11 billion [245]. As the demand for orthopedic surgery continues to rise at an accelerated rate [246], a future infection-related healthcare and economic burden is anticipated [247,248,249,250]. Efficacious strategies to prevent and treat PJI, osteomyelitis, and its recurrence remain elusive, and innovative approaches are needed, particularly within the context of the continuous emergence of antimicrobial resistance in common etiological agents of PJI and osteomyelitis. Common pathogens associated with implants and osteomyelitis include *Staphylococcus aureus*, *Staphylococcus epidermidis*, *Escherichia coli*, and *Pseudomonas aeruginosa* [251,252,253]. Here, we discuss the potential role of sphingolipids as a promising approach to combat orthopedic infection.

### 3.1. The Antibacterial Activity of Sphingolipids

Sphingolipids located within the stratum corneum of the skin exhibit antimicrobial activity against both Gram-positive and Gram-negative bacteria and likely contribute to the permeability and innate immunologic barriers of the skin [254,255,256,257]. Included among these lipids are free sphingosine, phytosphingosine, and dihydrosphingosine derived from epithelial sphingolipids and via the activation of hydrolytic enzymes. Sphingosine, phytosphingosine, and dihydrosphingosine deliver varying degrees of antimicrobial activity to many pathogens, including *S. aureus*, *E. coli*, and *P. aeruginosa.* However, the bactericidal mechanisms that underpin these properties remain poorly understood, and despite a 2019 review by Kunz et al. [258] hypothesizing an association with the Gram classification, this remains to be fully elucidated [259,260,261]. A study by Bibel et al. [261] suggested the site of activity was the cell wall. Here, electron microscopy revealed that supplementation of *S. aureus* with sphingolipids introduced cell wall lesions to the bacterial cell, disrupting the membrane and leading to release of its intracellular contents. Fischer et al. [256] also demonstrated that sphingolipids induced differential ultrastructural damage, accumulated within *S. aureus*, and induced intracellular inclusions. Further, it has been reported that bactericidal sphingosine inhibited the adherence of *Streptococcus mutans* onto a hydroxyapatite-coated surface and disrupted pre-formed biofilm, suggesting a role in the control of oral biofilms [262].

In respiratory epithelia, it has been shown that the increased abundance of ceramides in people with cystic fibrosis (CF) relative to those without contributes to susceptibility to endobronchial *P. aeruginosa* infection—a clinical presentation underpinned by inadequate acid ceramidase-coding *ASAH1* gene expression and subsequent ceramide accumulation, and deficient upregulation of bactericidal sphingosine production in the presence of *P. aeruginosa* [263]. This imbalance was ameliorated following treatment with recombinant human acid ceramidase (rhAC), which attenuated the aberrant inflammation previously observed within the CF population in addition to increasing the proportion of sphingosine within the host plasma membrane, reducing the adherence of both *S. aureus* and *P. aeruginosa* to the apical membrane of host epithelial cells [263].

In addition to the direct interactions of host sphingolipids and bacteria, it has been suggested that the generation of ROS during the cleavage of sphingomyelin into ceramide and phosphorylcholine facilitates the apoptosis of macrophages infected with *P. aeruginosa* [264]. Thus, the interaction of host sphingolipids with the wider inflammasome provides an environment conducive to the clearance of pathogenic bacteria in both human and murine models. Furthermore, host glycosphingolipid β-glucosylceramide, which acts as a damage-associated molecular pattern and is the only identified ligand of macrophage-inducible C-type lectin, is associated with the formation of neutrophil extracellular traps, thus facilitating bacterial clearance by host neutrophils [265].

### 3.2. Sphingolipid-Mediated Host Cell Invasion

**Figure 5:**
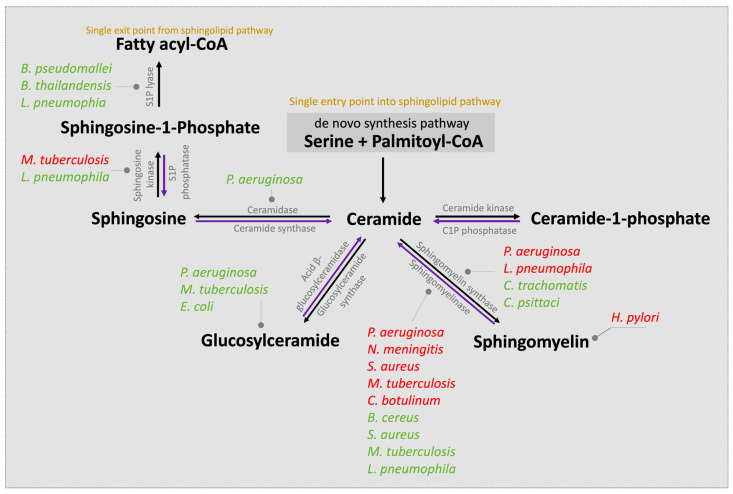
Pathogenic bacteria are able to adhere, engage, enter, and hijack host cell responses via the sphingolipid pathway. In addition to *S. aureus*, *P. aeruginosa*, and *E. coli*, *Helicobacter pylor*i [274], *Neisseria meningitis* [275,276], *Clostridium botulinum* [277,278], *Mycobacterium tuberculosis* [279,280], *Chlamydia psittaci* [281], *Bacillus cereus* [282], *Burkolderia pseudomallei* and *Burkholderia thailandensis* [283,284], and *Legionella pneumophila* [285] are pathogens able to infect bone or that have been reported within the bone marrow. *Chlamydia trochomatis* is a notorious pathogen able to avoid destruction and persist within host cells [286], and is associated with reactive RA [287]. These bacteria target host cell sphingolipid enzymes either directly (red) or indirectly (green). Image adapted from Rolando et al. [266].

Most bacteria do not contain sphingolipids, but some have evolved mechanisms to utilize the sphingolipids produced by eukaryotic cells to promote their own virulence (Figure 5). To this end, and to ensure their own survival, pathogenic bacteria have evolved many strategies to adhere, engage, enter, and hijack host cell responses. To successfully invade host cells, pathogenic bacteria modulate host cell membrane properties, and the manipulation of lipid biogenesis and membrane stability via the sphingolipid pathway is emerging as a primary pathway in bacterial host cell control [266]. As it participates in membrane reorganization and the formation of ceramide-enriched platforms, a frequent target by pathogens is aSMase. As described, sphingosine displays antibacterial properties, and thus, decreasing sphingosine levels indirectly by activating aSMase is beneficial for pathogen survival and can occur within a few minutes after infection [267]. For example, *P. aeruginosa* has been shown to stimulate aSMase formation within the plasma membrane, which leads to the production and release of ceramide within sphingolipid-rich rafts [267]. Although sphingolipid-enriched rafts are essential sites of origin for cell-mediated signaling and homeostasis [268], the newly aSMase-induced ceramide subsequently reorganizes these rafts into larger ceramide-enriched signaling macrodomains or platforms. These binding platforms are used by *P. aeruginosa* to enter and internalize within the host cell, inducing apoptosis and regulating the cytokine response. In a murine-based study, the authors speculated that the ceramide-enriched platforms may trap receptor molecules (e.g., cystic fibrosis transmembrane conductance regulator (CFTR) and CD95), thereby biophysically inhibiting the host cell defense signaling mechanism that would otherwise respond by excluding interactions with the bacterium [268]. The formation of ceramide-enriched platforms has also been reported to induce the local accumulation of β1-integrins, which subsequently suppresses acid ceramidase activity, thereby leading to the additional accumulation of ceramide, which, in parallel, reduces beneficial and antibacterial surface levels of sphingosine [269]. Notably, *S. aureus* [270] and *Clostridium difficile* [271] infections also activate aSMase and are essential for infection, while SM is required for the entry of *Helicobacter pylori* [272]. Further, host cell glycosphingolipids are also reported to contribute to the adhesion of *P. aeruginosa* and are critical for the internalization of *P. aeruginosa* into nonphagocytic cells [273].

An efficient host response to bacterial invasion is the fusion of late phagosomes and lysosomes, a process critical for the relocation of antibacterial lysosomal hydrolases into phagosomes and thereby facilitating the transport and elimination of pathogens from the host cell [288,289]. The role of aSMase in the efficient fusion and formation of phagolysosomes is essential [290]. Li et al. [291] showed that in *S. aureus*, ROS-induced aSMase formation via CD44 resulted in ceramide release, the clustering of CD44 within ceramide-enriched membrane platforms, and a rapid rearrangement of the actin cytoskeleton with cortical actin polymerization. Notably, aSMase-deficient cells abrogated these signaling events and reduced the formation of phagolysosomes and the internalization of *S. aureus* into macrophages by 60–80%. This thereby facilitated *S. aureus* survival and replication, and increased the severity of infection in vivo. Similar to *P. aeruginosa*, this suggests an essential role of aSMase in not only promoting ceramide-enriched rafts and entry into the cell but also in assisting *S. aureus* by disrupting its transport and elimination within host cell phagolysosomes.

Although their study regards a unicellular eukaryote and not a prokaryote, Ghosh et al. [292] revealed that an increase in intracellular de novo ceramide synthesis and activation of aSMase led to a decrease in the growth and survival of the obligate intracellular protozoan *Leishmania donovani*. The study reported that enhanced levels of ceramide within murine peritoneal macrophages were essential for the downregulation of classical protein kinase C (PKC) activity and the upregulation of calcium-independent atypical PKC-ζ expression. Further, increased ceramide impaired the phosphorylation of MAPK and suppressed the formation of reactive nitrogen species (RNS) within host macrophages, which further facilitated the survival of this parasite. Immune cells deficient in aSMase have been reported to produce RNS but were nevertheless incapable of inhibiting the intracellular proliferation of prokaryotic *Listeria monocytogenes* in vitro [293]. Therefore, this may suggest the limited role of RNS during pathogen invasion and further highlights the importance of aSMase and ceramides in pathogen control and infection. Together, these studies show that during severe infection and sepsis, the stress responsive enzyme aSMase is activated, and the hydrolysis and degradation of, for example, relatively inert SM into ceramide, together with enhanced de novo ceramide synthesis, unfastens the doorway for invading pathogens.

*P. aeruginosa* also produces and secretes sphingolipid-metabolizing enzymes. The large extracellular protease hemolytic phospholipase C (PlcH) is known to be a virulence determinant in animal models of infection [294]. Multifunctional PlcH hydrolyzes phosphatidylcholine and SM to produce diacylglycerol and ceramide, respectively, lysing erythrocyte membranes and causing severe toxicity and damage to host cells [295,296,297]. *P. aeruginosa* has been shown to secrete a neutral ceramidase capable of cleaving the *N*-acyl linkage between sphingosine and the fatty acids of ceramide [298,299]. Notably, ceramidase-deficient *P. aeruginosa* attenuated PlcH-induced hemolysis, confirming the primary role of bacterial ceramidase and PlcH in virulence in vitro. Further, a PlcH-mutant murine model investigated by Kida et al. [300] displayed decreased *P. aeruginosa* virulence, thereby confirming that bacteria utilize sphingolipid-mediated PlcH to contribute to virulence in vivo. Host-derived sphingolipids can also induce and enhance the secretion of a neutral *P. aeruginosa* ceramidase, which serves to further enhance PlcH-mediated hemolysis and cytotoxicity via the *P. aeruginosa* sphingosine (sph)-inducible transcriptional regulator *sphR* [298,299]. To this end, a mobility shift assay demonstrated that *P. aeruginosa*-located SphR specifically bound free sphingoid bases such as sphingosine, phytosphingosine, and dihydrosphingosine but not SM or ceramide. Further, in vitro data have shown that deletion of *sphR* is toxic for bacteria and increases their sensitivity to the antimicrobial effects of sphingosine. In support of this, the deletion of *sphR* resulted in reduced *P. aeruginos*a survival in vivo [301]. Together, this shows that *P. aeruginosa* is capable of intercepting and responding to host immune signaling molecules via their own sphingolipid-metabolizing enzymes and raises the question of whether sphingolipids and, in particular sphingosine, as well as SphR-inhibitor therapy, may contribute an important role in controlling virulence and infection.

Further to the above pathogen interactions with host sphingolipids, a growing body of evidence has identified several bacterial species capable of endogenous sphingolipid biosynthesis. Recent studies have demonstrated that several Gram-negative bacterial species are capable of synthesizing sphingolipids. A 2022 study by Stankevibiute et al. [302] identified *Stretomyces aurantiacus* as the first known Gram-positive bacterial species to possess a complete and functional sphingolipid biosynthetic pathway, likely acquired by horizontal gene transfer from Gram-negative Deltaproteobacteria. Bacterial sphingolipids differ structurally from their eukaryotic counterparts, likely as a factor of limited synthetic enzyme homology between the prokaryotic and eukaryotic domains. Additionally, biochemical research has revealed that the biosynthetic process occurs in different sequences between both domains, with further phylogenetic analyses concluding that mechanisms of ceramide synthesis in bacteria and eukaryotes are the products of convergent evolution [302].

### 3.3. Sphingolipids, Bacterial Inflammation, and Bone Loss

Host–pathogen interaction triggers excessive inflammation via the orchestrated release of cytokines, including IL-1β, IL-6, IL-8, and TNFα, which subsequently impairs tissue integrity [303,304]. Although highly complex, the simultaneous release and crosstalk of anti-inflammatory cytokines (e.g., IL-10) acts to counterbalance and compartmentalize the inflammatory response, reducing host tissue damage and facilitating host cell survival [305]. The extent and magnitude of the inflammatory response is related to disease severity and clinical outcomes [306,307,308]. During this inflammatory pathogenesis process, bacterial pathogens activate several cellular signaling cascades, including RANK/RANKL signaling pathways (e.g., NF-κB, MAPK, and PI3K), where upregulation activates osteoclastogenesis and, ultimately, bone resorption [309]. Monocytes and macrophages are osteoclast precursor cells and fuse to form osteoclasts, thereby contributing a critical role in facilitating bone resorption [310]. Furthermore, *Porphymonas gingivalis*-synthesized phosphoglycerol dihydroceramides were found to interact with non-muscle myosin IIA (Myh9), a host cytoplasmic osteoclast cell fusion-regulatory protein that generates Ras-related C3 botulinum toxin substrate 1 (Rac1) and subsequently upregulates the expression of the osteoclast fusogen dendritic cell-specific transmembrane protein (DC-STAMP). The synergistic effect of this upregulation in osteoclast fusion, paired with the inhibitory effect of *P. gingivalis* phosphoethanolamine dihydroceramides and phosphoglycerol dihydroceramides on osteoblast differentiation gene expression, has been shown to facilitate the alveolar bone loss observed in *P. gingivalis*-associated periodontitis. The associated mechanistic details of these virulence factors have been succinctly reviewed by Olsen and Nichols [311].

S1PR_2_ has been shown to play an essential role in modulating bacterial-induced proinflammatory cytokine release via PI3K, extracellular signal-regulated kinase, c-Jun N-terminal kinase, and NF-κB pathways [312], and also to regulate RANKL-induced osteoclastogenesis [313]. The authors showed that the inhibition of S1PR_2_ reduced IL-1β, TNFα, IL-6, and S1P production within murine bone marrow cells following exposure to *Aggregatobacter actinomycetemcomitans*; inhibited infection-induced bone marrow-derived monocyte and macrophage chemotaxis; and suppressed RANKL-induced bone resorption. It was concluded that the downregulation of S1PR_2_ signaling may be a novel therapeutic strategy to treat inflammatory bone loss diseases.

There is evidence showing the IL-1-induced promotion of de novo ceramide biosynthesis via SPT during an acute-phase infection response [314,315]. Further, increased ceramide production is reported to induce IL-1β and TNFα secretion by macrophages [316] and also lead to macrophage death [317]. *S. aureus* infection has also been shown to increase ceramide levels, which stimulate IL-1β and TNFα signaling via the release of cathepsin B and D from lysosomes. This potentially occurs through the binding of its major toxin, α-toxin, and subsequent activation of aSMase [318]. Notably, mice challenged with endotoxin generated a 2-fold increase in aSMase plasma levels and the release of pro-inflammatory cytokines [319]. Similarly, increased ceramide content within serum lipoproteins alongside a 10-fold increase in the basal level of circulating aSMase for up to 24 h was reported following LPS administration in mice [314]. Further, the study also showed that aSMase knockout mice retained the LPS-induced increase in serum ceramide, suggesting the compensatory activation of de novo ceramide synthesis within the liver. Alterations in ceramide and C1P levels have also been shown to modulate the direction of the inflammatory cascade via a TNFα-controlled response to LPS, a constituent of the cell walls of Gram-negative bacteria [320]. Finally, ceramides modulate the efficacy of macrophages to sense and respond to microbes via pattern recognition receptors, including toll-like receptors [321]. To this end, ceramide accumulation has been described as a critical component in mediating various immune cell functions, including the tailored regulation of the cell response to bacteria and other foreign invaders [322]. Further, Albeituni and Stiban [322] speculated that the ability of ceramides to induce downstream signaling for the production of proinflammatory cytokines during an infection allows macrophages to persist and orchestrate the activation of immune cells that subsequently produce survival factors, including macrophage-colony stimulating factor. Following successful pathogen clearance, ceramides subsequently assist in mediating macrophage apoptosis during the downregulation of immune cell activation and likely promoting healthy bone cell function.

IL-1β is central to the lethal effect of *P. aeruginosa* infection, and Grassmé et al. [267] showed that sphingolipid-enriched platforms are important for stabilizing IL-1β levels and likely other cytokines during infection. Both sphingosine and S1P also confer key roles in *P. aeruginosa*-induced inflammatory lung injury via SPHK2. Upon infection and mediated by PKC-ζ, SPHK2 is phosphorylated, increasing its nuclear localization and thereby upregulating S1P and increasing histone acetylation and IL-6 and TNFα secretion [323]. The study indicates a critical role of nuclear SPHK2/S1P signaling in epigenetic regulation of *P. aeruginosa*-mediated inflammatory lung injury, and targeting SPHK2 may represent a potential strategy to reduce lung inflammation. Although not an orthopedic application, a murine model of CF, a condition commonly challenged by *P. aeruginosa*, displayed a significant reduction in sphingosine alongside ceramide accumulation within the respiratory tract due to a suppression of aSMase activity [324]. Remarkably, inhalation with sphingosine, FTY720, or aSMase rescued susceptible mice from infection, and the authors suggested the use of sphingosine as a promising novel approach to prevent *P. aeruginosa* infection. Further, Tabazavareh et al. [325] demonstrated that CF mice were highly susceptible to *S. aureus* and MRSA infection compared to wild-type mice and that inhalation of sphingosine for 30–40 min before introducing the pathogen significantly reduced the incidence of pulmonary infection by these pathogens. Notably, the study also confirmed the bactericidal activity of sphingosine on *S. aureus* cells in vitro and showed that the inclusion of ceramide abrogated this effect. Seitz and colleagues investigated sphingosine and phytosphingosine coatings on endotracheal ventilation tubes and demonstrated that the coatings prevented pathogen surface adherence; facilitated the immediate killing of *P. aeruginosa*, *Acinetobater baumannii*, and *S. aureus* species; and prevented biofilm formation. Notably, sphingosine conferred no obvious side effects to tracheal epithelial cells, and importantly, the application of a sphingosine coating prevented *P. aeruginosa* and *S. aureus* pneumonia in vivo [326]. Beck et al. [327] demonstrated the bactericidal effect of sphingosine against three different strains of *S. epidermidis*, which represented frequent microbes involved in PJI. The study showed that coating a titanium K-wire with sphingosine prevented planktonic surface contamination and reduced biofilm formation by 99.942%. Further, the application of a sphingosine solution (1 mM) on preformed biofilm eliminated 99.999% of bacteria on titanium surfaces as well as on steel and polymethylmethacrylate surfaces.

## 4. Sphingolipids, Viral Host Cell Entry, and Orthopedic Infection

The impact and challenges associated with bacterial and, more rarely, fungal PJIs, are well recognized [328]. However, new and emerging studies emphasize the important need to investigate viral musculoskeletal infections, with concerns that their influence may be underestimated [329,330]. Genomic and sequencing analyses reveal the presence of viral DNA and RNA within bone tissue, but little is understood about the interaction, persistence, and pathogenesis of viruses in relation to bone disease [331]. Recent evidence suggests that viruses can cause moderate to severe arthritis and osteitis, with risk factors such as pre-existing rheumatologic disease contributing to higher disease severity and duration of symptoms [329,330]. To this end, viral agents such as human immunodeficiency virus (HIV), hepatitis B, hepatitis C, and alphavirus, among others, have been shown to cause viral arthritis [332]. Typically, viral arthritis has been interpreted as viral-induced autoimmunity and a resulting inflammatory response, rather than the result of direct viral infection with primary damage to bone and cartilage tissue [330]. However, in the case of alphavirus, direct osteoblastic infection and subsequent disruption of RANKL/OPG expression has been identified [333]. Further, Dimitriou et al. [334] recently reported the significantly increased risk of PJI in HIV-positive patients. Notably, HIV-1 directly infected osteoclasts, causing increased adhesion and osteolytic activity and leading to bone resorption and defect formation [335]. This appeared to occur via modification of the structure of the sealing zone and thus altered the resorption machinery of the osteoclast. Further, a virally induced decrease in osteoblast-related alkaline phosphatase expression was also measured. Similarly, a recent study by Haudenschild et al. [336] provided the first evidence that SARS-CoV-2 infection led to acute bone loss, increased osteoclast numbers, and thinner growth plates. This was speculated to occur via hyperinflammation and decreased mobility together with the direct effects of SARS-CoV-2 infection of bone cells. The study concluded that the bone loss measured may decrease whole-bone mechanical strength and increase the risk of fragility fractures, particularly in older patients.

Sphingolipids play an important role in the viral infection process [337], including (i) viral attachment and entry into the host cell plasma membrane. This is predominantly influenced by sphingolipid-enriched membrane microdomains where structural alterations influence and modify the fusion of the host plasma membrane with the viral membrane. These first interactions between viruses and cells subsequently effect biophysical processes such as (ii) endocytosis, where the endocytosis of viruses and their subsequent uptake and intracellular trafficking are modulated. Similar to bacteria, this occurs via the formation of condensed ceramide-enriched membrane platforms in response to sphingomyelinase activation or ceramide inhibition [338,339]. Thus, conditions that favor microdomain formation enhance viral infection. Finally, (iii) sphingolipids are involved in changes in host signaling and cellular metabolism, which directly influences the viral replication cycle [337]. Sphingomyelin and aSMase have been implicated in the early steps towards the adhesion of Ebola virus, rhinovirus, measles viruses, Japanese encephalitis virus, and SARS-CoV-2 to host cells [337,340,341,342]. Sphingomyelin was shown to be required for attachment, while aSMase was strongly associated with the interaction of viral particles on the host membrane. Evidence of a protective role of ceramides has been shown, where, for example, active influenza A virus replication resulted in a significant increase in ceramide generation via the de novo biosynthesis pathway [343]. Notably, this did not occur when a heat-inactivated virus was investigated. More thorough reviews of the viral response to sphingolipids have been published [337,344,345,346], and although much remains to be discovered, the targeting of host cell sphingolipid metabolism to limit viral adhesion, entry, trafficking, and replication within host cells may offer exciting future therapeutic avenues of exploitation to aid in addressing the challenges associated with orthopedic PJI.

## 5. Discussion

Regulation of the diverse sphingolipid machinery is tightly governed by the highly conserved nature of their synthesis, degradation, and modification. This review suggests an extremely complex sphingolipidome where the targeted action of each sphingolipid will depend on changes in the expression of many of the other bioactive sphingolipids present. The intricacy of this interconnected network remains to be fully understood but is considered sophisticated and convoluted, as, for example, an increased flux through the de novo pathway does not necessarily lead to ceramide accumulation unless its conversion to, e.g., SM, C1P, or glucosylceramide is also suppressed or impaired [6]. As such, the downregulation or complete inhibition of one bioactive metabolite may have varying levels of effects in terms of ultimately promoting beneficial bone tissue regeneration or, indeed, dysfunction. Furthermore, this review highlights the dynamic adaptability of this system, as, for example, when aSMase levels are pharmacologically or pathogenically inhibited, a compensatory route is activated where ceramide levels are generated via the de novo synthesis route to overcome this loss. Although under healthy bone conditions this is undoubtedly important for ensuring the critical steady state of physiological levels of ceramide, this pathway also opens the door for dysregulation during the pathogenesis of various metabolic disorders. Several bacterial pathogens actively modulate the sphingolipid machinery in host cells to promote colonization, and similarly, it is increasingly clear that sphingolipids also contribute a dual role in both assisting with the establishment of infection while also activating host defenses against the invading pathogen. Further, it is conceivable that alterations in these bioactive metabolites are not the cause but rather a symptom of dysfunctional sphingolipid metabolism, which ultimately leads to the pathophysiologies described.

In terms of their mechanism of action and co-dependent activity, much remains to be resolved. For example, five distinct ceramidases and five differing sphingomyelinases that localize to the plasma membrane, ER, Golgi complex, lysosome, mitochondria, or extracellular space have been identified. Six distinguishable ceramide synthases, which show distinct preference for interacting with varying FAs, also contribute to this complexity [6]. An additional tier of intricacy is the recent discovery of several atypical sphingolipids. For example, 1-deoxysphingolipids are formed via the alternate action of SPT on alanine instead of the substrate serine [347] in both eukaryotes and prokaryotes [348]. Further, and as they lack the essential C1-hydroxyl group, 1-deoxysphingolipids cannot be converted to complex sphingolipids and are not degraded via the canonical catabolic pathways, thereby undergoing repeated de- and re-acylation cycles. Thus, and due to their elevated cytotoxicity compared to canonical sphingolipids, pathologically increased levels of 1-deoxysphingolipids are involved in several disease conditions, including type 2 diabetes and sensory and autonomic neuropathy [347,349,350,351].

## 6. Conclusions

In conclusion, dysregulation within this important group of metabolites appears to be central to many chronic metabolic diseases and disorders where unmet orthopedic clinical needs remain. For example, the discovery of new technologies and approaches are urgently needed to limit bone loss and damage due to age-induced osteoporosis, rheumatoid and osteoarthritis, and radiotherapy. This is of high significance, as our aging population continues to increase globally, and the treatment and care of conditions such as osteoporosis, osteoarthritis, and cancer will undoubtedly bear a huge health–economic burden in all regions of the world [84,85,86]. Finally, the emergence of antibiotic-resistant bacterial strains is also driving the innovation and development of next-generation approaches to resolving the contemporary issues associated with orthopedic PJI and osteomyelitis. This review suggests the pivotal role of sphingolipids in not only modulating host cell–pathogen interactions but also in regulating inflammation, osteoclastic activity, and bone resorption during infection. As such, the future use of sphingolipid therapy is highly promising; however, many open questions remain, and a greater understanding of the multifaceted sphingolipidome may represent the next frontier in this regard.

## Data Availability

Data is contained within the article.
